# The relationship between monocyte to high-density lipoprotein cholesterol ratio and diabetic nephropathy

**DOI:** 10.12669/pjms.35.4.534

**Published:** 2019

**Authors:** Erhan Onalan

**Affiliations:** Erhan Onalan, Department of Internal Medicine, Faculty of Medicine, Firat University, 23000, Elazig, Turkey

**Keywords:** Diabetes Mellitus, Diabetic Nephropathy, Monocyte count to high-density lipoprotein cholesterol ratio

## Abstract

**Objective:**

This study aims to investigate the relationship of monocyte to high-density lipoprotein cholesterol ratio (MHR) with diabetes mellitus (DM) and diabetic nephropathy.

**Methods:**

This study included 262 Type-2 diabetes mellitus patients, of which 60 had diabetic nephropathy and 202 did not have diabetic nephropathy who presented to the internal diseases polyclinic at Firat University Medical Faculty Hospital between May 2018 and October 2018 and 50 healthy control subjects. A retrospective scan of patient files was conducted and information relevant to nephropathy such as hemoglobin, glycated hemoglobin levels (HbA1c), hematocrit count (HCT), monocyte count, LDL, HDL, triglyceride levels, and microvascular complications were acquired.

**Results:**

We determined MHR values as 11.9±5.5 and 8.4±2.9 respectively for the diabetic and healthy groups. There was a statistically significant difference between the two groups in terms of MHR, with a positive correlation between diabetes and MHR (< 0.001; r: 0.241). Moreover, glucose, HDL, and triglyceride levels were different between the two groups with statistical significance (respectively, p< 0.001; p< 0.001; p< 0.001). Our study found higher MHR levels for patients with diabetic nephropathy compared to those without diabetic nephropathy (respectively, 17.1±7.9 and 10.3±3.3) and determined statistical significance and a negative correlation (p< 0.001; r: -0.512).

**Conclusion:**

Our results suggest that an elevated MHR can be a biomarker for diabetic nephropathy, allowing the detection of diabetic nephropathy with simple and inexpensive laboratory tests.

## INTRODUCTION

Diabetes mellitus is a systemic chronic disease of the metabolism that progresses with chronic hyperglycemia. It is characterized by disturbances in carbohydrate, protein, and lipid metabolisms that appear due to a partial or complete deficiency of insulin and/or insulin resistance. In addition to the acute metabolic complications, hyperglycemia associated with DM can lead to damage and dysfunction in certain organs and systems of the body in the long term; particularly the eyes, kidneys, heart, and blood vessels.^1^

Diabetic nephropathy currently ranks first among factors that lead to end-stage renal disease (ESRD) in the world due to the significant increase in the prevalence rates of diabetes and associated complications. It is well-known that it triggers microvascular complications, both through the direct effect of hyperglycemia and by causing local and systemic increases in various cytokines, chemokines, and growth factors. While hyperglycemia is the main factor that initiates the pathological process at the early stages of diabetic nephropathy, hypertension (HT) significantly accelerates this process at later stages. As the disease can progress through various stages and reach end-stage renal disease (ESRD) via an uneventful course, it is recommended that Type-2 diabetes patients are screened for microalbuminuria starting from the time of diagnosis, and that Type-1 diabetics are screened after a five-year period on average. At the early stages of diabetes preceding the appearance of morphological changes, certain changes such as an increased renal plasma flow, intraglomerular hydrostatic pressure, and glomerular filtration rate become manifest.^2^ These changes may appear due to a wide variety of factors that result from high glucose-induced interactions between a group of metabolic and hormonal. It was shown that loss of sulfated proteoglycans and anionic regions in the basal membrane of the glomerulus and the mesangial matrix caused excess accumulation of proteoglycans such as chondroitin sulphate and dermatan sulphate in these areas.^3^ This causes a decrease in charge-dependent renal selectivity and an increase in the thickness of the basal membrane. In tissues where glucose intake is independent of insulin, excess glucose is typically metabolized to sorbitol through the polyol pathway and this reaction is catalyzed by the aldose reductase enzyme. Many experimental diabetes models have shown this pathway to be important for the development of microvascular complications and to be intercepted by inhibitors of the aldose reductase enzyme in.^4^

Monocyte to high-density lipoprotein (HDL) ratio (MHR) indicates inflammation and oxidative stress based on the anti-inflammatory and antioxidant effects of high-density lipoprotein cholesterol (HDL-C), as well as the proinflammatory effect of monocytes. Various studies have used these measurements to determine whether inflammation and atherosclerosis have contributed to the etiopathogenesis of cardiovascular and cerebrovascular diseases.^5^-^12^ Although many studies have investigated MHR and inflammatory processes in particular, to our knowledge, there are not adequate studies on MHR and DN. This study aimed to address this reality of the literature by investigating the relationship between monocyte to high-density lipoprotein cholesterol ratio (MHR) and decreased renal function in patients with diabetic nephropathy.

## METHODS

This study was approved by the Firat University Scientific Research Projects Coordination Unit on 22.1.2018, with the reference number 08. The data were composed based on a retrospective scan of the files of patients who presented to the internal diseases clinic and polyclinic at Firat University Medical Faculty Hospital between May 2018 and October 2018. The study included 262 diabetic patients, of which 60 had diabetic nephropathy and a control group comprised of 50 healthy individuals. Patients with diabetes and healthy individuals were assigned to two separate groups. The diabetic patient group consisted of patients aged between 30-75 who did not have any other diseases and had presented to the internal diseases polyclinic for diabetes. Patients with another chronic disease (coronary artery disease, hematological diseases, malignancies, severe liver disease, severe kidney failure), a diagnosis of rheumatological diseases or infectious diseases (tuberculosis, malaria, Brucella) that progress with inflammation causing an elevated monocyte count, renal diseases besides type 2 diabetes mellitus that cause proteinuria, a diagnosis of type 1 diabetes mellitus, and active psychiatric disorders were excluded from the study. A retrospective scan of files was conducted and information relevant to nephropathy such as hemoglobin levels, glycated hemoglobin levels (HbA1c), hematocrit count (hct), monocyte count, LDL, HDL, triglyceride levels, and microvascular complications were obtained. Diabetic patients with + proteinuria and/or creatinine levels above 1.2 mg/dl in complete urinalysis were considered cases of diabetic nephropathy. Estimated GFR (eGFR: estimated Glomerular Filtration Rate) was calculated using the Modification of Diet in Renal Disease (MDRD) equation.^13^ The MDRD GFR equation estimates glomerular filtration rate based on creatinine and patient characteristics. Demographic information (age, gender) was obtained through the scan of polyclinic patient files.

### Statistical Analysis

All statistical analyses were performed using a computer packaged software program (SPSS-22). In addition to descriptive statistical methods [Mean (X̄), Standard deviation (SD)]; quantitative data was analyzed using the Student’s t-test in testing parameters that show normal distribution and one-way variance analysis in comparisons across groups (One-way ANOVA). The Wilcoxon matched pairs test was utilized to assess the significance of the difference between pairs and the chi-square test was used for the comparison of qualitative data. The results were evaluated with a 95% confidence interval and a p<0.05 level of significance.

## RESULTS

We retrospectively compared the demographic data and laboratory parameters of 262 diabetic patients, which included 60 patients with diabetic nephropathy with those of 50 healthy individuals. Table-I presents the comparison of the diabetic and healthy groups with regard to their demographic data and laboratory parameters. While the mean age of the diabetic group was 57.8±10 years, the mean age of the healthy group was 37.1±12.4 years. We determined the MHR respectively as 11.9±5.5 and 8.4±2.9 for the diabetic group and the healthy group. There was a statistically significant difference between the two groups in terms of MHR, with a positive correlation between diabetes and MHR (< 0.001; r: 0.241). Moreover, glucose, HDL, and triglyceride levels were different between the two groups with statistical significance (respectively, p< 0.001; p< 0.001; p< 0.001).

**Table I:** Demographic and laboratory data of diabetic and healthy individuals.

	Groups	N	Mean	Standard Deviation	p-value
Age(years)	Healthy	50	37.1	12.4	< 0.001
	Diabetic	262	57.8	10	
Glucose(mg/dL)	Healthy	50	98.2	8.1	< 0.001
	Diabetic	262	262.9	87.9	
LDL(mg/dL)	Healthy	50	119.8	34.3	0.08
	Diabetic	262	131.1	38.9	
HDL(mg/dL)	Healthy	50	49.5	11.1	< 0.001
	Diabetic	262	38.4	17.9	
TG(mg/dL)	Healthy	50	101.9	41.1	< 0.001
	Diabetic	262	217.4	128.9	
Monocyte(x109/L)	Healthy	50	397.6	90.6	0.5
	Diabetic	262	408.8	112.3	
Creatinine(mg/dL)	Healthy	50	0.78	0.17	0.2
	Diabetic	262	0.89	0.71	
MHR	Healthy	50	8.4	2.9	< 0.001
	Diabetic	262	11.9	5.5	
eGFR(mL/min per 1.73m2)	Healthy	50	103.9	10.9	< 0.001
	Diabetic	262	77.9	14.1	

LDL: Low density lipoprotein, TG: Triglyceride, HDL: High Density Lipoprotein, MHR: Monocyte to high-density lipoprotein cholesterol ratio, eGFR: Estimated Glomerular Filtration Rate

**Fig. 1:**
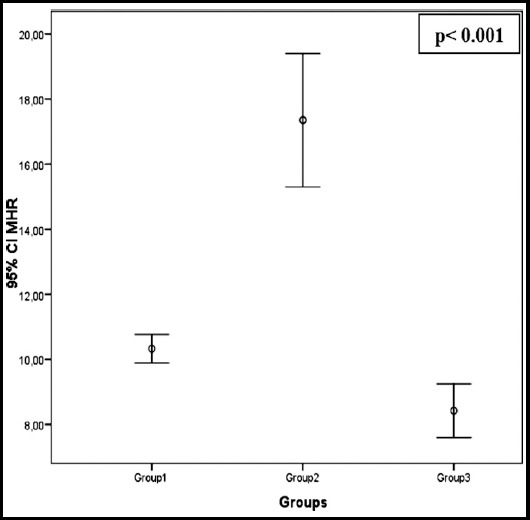
Comparison of MHR between group1(diabetic patients without nephropathy), group2(diabetic patients with nephropathy) and group3(control group comprised of healthy individuals).

The demographic data and laboratory values of patients with and without diabetic nephropathy and the relationship between these groups are shown in Table-II. Our study found higher MHR levels for patients with diabetic nephropathy compared to those without diabetic nephropathy (respectively, 17.1±7.9 and 10.3±3.3) and determined statistical significance and a negative correlation (p< 0.001; r: -0.512).

**Table II:** Demographic and laboratory data of patients with and without diabetic nephropathy.

	Groups	N	Mean	Standard Deviation	p-value
Age(years)	DM+DN	60	54.8	10.6	< 0.001
	DM	202	58.7	9.6	
HbA1c(%)	DM+DN	60	10.4	2.4	< 0.001
	DM	202	7.7	1.4	
Glucose(mg/dL)	DM+DN	60	322.7	117.3	< 0.001
	DM	202	245.2	67	
LDL(mg/dL)	DM+DN	60	122.5	42.2	0.08
	DM	202	132.3	37.6	
HDL(mg/dL)	DM+DN	60	33.8	35.2	< 0.001
	DM	202	39.8	6.9	
TG(mg/dL)	DM+DN	60	246.6	142.3	< 0.05
	DM	202	208.7	123.7	
Monocyte(x109/L)	DM+DN	60	429.3	121.7	0.1
	DM	202	402.7	108.9	
Creatinine(mg/dl)	DM+DN	60	1.25	1.41	< 0.001
	DM	202	0.79	0.17	
MHR	DM+DN	60	17.1	7.9	< 0.001
	DM	202	10.3	3.3	
eGFR(mL/min per 1.73m2)	DM+DN	60	80.5	8.7	< 0.05
	DM	202	76.3	16.4	

LDL: Low density lipoprotein, TG: Triglyceride, HDL: High Density Lipoprotein, MHR: Monocyte to high-density lipoprotein cholesterol ratio, eGFR: Estimated Glomerular Filtration Rate

## DISCUSSION

This study showed that the increase in the MHR of patients with diabetic nephropathy was correlated with an increase in 24-hour urine protein levels. It compared individuals with and without Type-2 diabetes and diabetic patients with and without diabetic nephropathy; and to our knowledge, it is the first study to demonstrate that those with diabetic nephropathy had higher MHR values compared to other diabetic patients without nephropathy and healthy controls.

Diabetes mellitus is a chronic disease of the metabolism that leads to disturbances in the carbohydrate, protein, and lipid metabolisms due to a partial or absolute deficiency in insulin hormone secretion and/or the effect of insulin. It is an important health problem with a prevalence that has been increasing throughout the world due to reasons such as a sedentary lifestyle, poor diet, and being overweight. There exist more than 130 million individuals with DM in the world. It is estimated that this number will reach 333 million by 2025 in case that the precautions suggested by the International Diabetes Federation are not followed.^14^ Complications associated with DM can be categorized under two groups as microvascular and macrovascular complications. Microvascular complications include; retinopathy, nephropathy, and neuropathy. On the other hand, macrovascular complications are coronary heart disease, peripheral vascular disease, and cerebrovascular diseases. Patients have a longer lifetime as a result of the advances in the treatment of DM. However, the prevalence of diabetic nephropathy (DN) and end-stage renal disease (ESRD) have increased. Based on the records of the Turkish Society of Nephrology from 2013, DN accounts for 36% of ESRD cases.^15^ The risk of developing DN has increased due to the increased survival times of Type-2 DM patients. The United Kingdom Prospective Diabetes Study (UKPDS) reported rates of microalbuminuria as 25%, macroalbuminuria as 5%, and elevated creatinine (above 2 mg/dl) or need for renal replacement therapy as 0.8% in patients with Type-2 DM at the 10^th^ year after diagnosis.^16^ The same study reported an annual progression rate of 2% for progression from normoalbuminuria to microalbuminuria, 2.8% for progression from microalbuminuria to macroalbuminuria, and 2.3% for progression from macroalbuminuria to elevated creatinine or starting dialysis.^16^

Independent risk factors for developing DN include hyperglycemia, hypertension, smoking, a high-protein diet, hyperlipidemia, being male (1.7 higher), being black, obesity, and genetic predisposition. The pathogenesis of DN implicates hemodynamic and metabolic factors triggered by hyperglycemia. Hemodynamically, renin-angiotensin-aldosterone (RAS) and endothelin systems are activated. The release of fibrotic cytokines such as transforming growth factor-beta (TGF-β) increases. The thicknesses of glomerular and tubular basal membranes and the Bowman’s capsule increase; podocyte damage occurs, and hyalinisation of afferent and efferent arterioles with hypertrophy in mesangial cells are encountered. As the glomerular mesangial volume increases, the surface area of glomerular capillaries and the Glomerular Filtration Rate (GFR) decrease, and diffuse glomerulosclerosis appears.^2^,^17^ The “Kimmelsteil-Wilson nodule,” which is pathognomonic in DN, is a form of nodular glomerulosclerosis that appears on the basis of diffuse glomerulosclerosis. Light microscopy reveals PAS positive and eosinophilic nodules with well-defined boundaries that originate from the mesangium and these have a prevalence of 12-46%.^3^

Monocytes and macrophages are cells that play an important role in the synthesis and release of proinflammatory and prooxidant cytokines.^18^ High-density lipoprotein cholesterol (HDL) was shown to protect the endothelium against the harmful effects of low-density lipoprotein cholesterol (LDL) and to prevent the oxidation of LDL cholesterol.^19^,^20^ In this way, HDL acts as an anti-inflammatory agent and an antioxidant.^21^ The high-density lipoprotein molecule demonstrates anti-inflammatory and antioxidant characteristics in various ways that include inhibiting the endothelial expression of adhesion proteins and the passage of monocytes in response to oxidised substances, as opposed to low-density lipoprotein (LDL), which enables the transport of oxidised molecules.^22^

Vural G. and colleagues compared patients with and without diabetic peripheral neuropathy and determined that patients with a high MHR were more likely to encounter polyeuropathy.^23^ In a study conducted by Ya G. and colleagues, MHR was stated to be strongly associated with coronary artery disease in diabetic patients and it was suggested that MHR cobe an important biomarker for predicting coronary artery disease in diabetic patients.^24^

In this study, we determined MHR values of 11.9±5.5 and 8.4±2.9 respectively for the diabetic and the healthy group. There was a statistically significant difference between the two groups in terms of MHR, with a positive correlation between diabetes and MHR. Moreover, glucose, HDL, and triglyceride levels were different between the two groups with statistical significance.

### Limitations of the study

The presented study was conducted on a retrospective basis and represented single-center experience.

## CONCLUSION

Our study found higher MHR levels for patients with diabetic nephropathy compared to those without diabetic nephropathy and determined statistical significance and a negative correlation. Our results have shown that an elevated MHR can be a biomarker for diabetic nephropathy, allowing the detection of diabetic nephropathy with simple and inexpensive laboratory tests.
